# *Ang-2* promotes lung cancer metastasis by increasing epithelial-mesenchymal transition

**DOI:** 10.18632/oncotarget.24061

**Published:** 2018-01-09

**Authors:** Zhizhen Dong, Jianrong Chen, Xuli Yang, Wenjie Zheng, Li Wang, Miao Fang, Mengna Wu, Min Yao, Dengfu Yao

**Affiliations:** ^1^ Research Center of Clinical Medicine, Affiliated Hospital of Nantong University, Nantong 226001, Jiangsu Province, China; ^2^ Department of Respiratory Medicine, Second Affiliated Hospital of Nantong University, Nantong 226001, Jiangsu Province, China; ^3^ Departments of Medical Informatics & Immunology, Medical School of Nantong University, Nantong 226001, Jiangsu Province, China

**Keywords:** lung cancer, Ang-2, RNA interference, EMT, prognosis

## Abstract

Lung cancer is the most common malignant tumor with increasing angiopoietin-2 (Ang-2) and a high rate of metastasis. However, the mechanism of Ang-2 enhancing tumor proliferation and facilitating metastasis remains to be clarified. In this study, Ang-2 expression and its gene transcription on effects of biological behaviors and epithelial-mesenchymal transition (EMT) were investigated in lung cancers. Total incidence of Ang-2 expression in the cancerous tissues was up to 91.8 % (112 of 122) with significantly higher (χ^2^=103.753, *P*^2^=7.883, *P*=0.005), differentiation degree (χ^2^=4.554, *P*=0.033), tumor node metastasis (TNM) staging (χ^2^=5.039, *P*=0.025), and 5-year survival rate (χ^2^ =11.220, *P*^2^=18.881, *P*^2^=0.81, *P*=0.776) or III & IV (χ^2^=1.845, *P*=0.174). Over-expression of Ang-2 or Ang-2 mRNA in lung A549 and NCI-H1975 cells were identified among different cell lines. When silencing *Ang-2* in A549 cells with specific shRNA-1 transfection, the cell proliferation was significantly inhibited in a time-dependent manner, with up-regulating E-cadherin, down-regulating Vimentin, Twist, and Snail expression, and decreasing invasion and metastasis of cancer cell abilities, suggesting that *Ang-2* promote tumor metastasis through increasing EMT, and it could be a potential target for lung cancer therapy.

## INTRODUCTION

Lung cancer is one of the most common malignancy tumors and the leading cause of cancer-related death [1, 2]. The early symptoms of patients with lung cancer are not obvious, with diagnosis difficult at early stage, tumor metastasis, drug resistance, and poor prognosis [3]. The occurrence and development of lung cancer is related to oncogene activation, tumor suppressor gene inactivation, protein functional effector sncRNAs (pfeRNAs), angiogenesis, and inflammation [4, 5]. Increasing evidence implicates that exosomes confer stability and can deliver their cargos such as proteins and nucleic acids to specific cell types, which subsequently serve as important messengers and carriers in lung carcinogenesis [6]. However, angiogenesis is a fundamental process involving a variety of pathological processes, sustains the progression of many neoplastic diseases, and successful establishment depends on a complex process of endothelial proliferation and organization [7, 8]. Moreover, it may enhance tumor cell proliferation and resistance to apoptosis, and facilitate metastasis. Angiopoietins (Angiopoietin-1, Ang-1 and Angiopoietin-2, Ang-2), Tie2 ligand-receptor, and vascular endothelial growth factor (VEGF)-mediated pathway are essential for the regulation of vascular maturation or stability, and have been implicated in the control of physiological angiogenesis [9, 10].

Recently, Ang-2 is an important proangiogenic factor that has been implicated in mediating inflammatory processes, and upregulated in multiple inflammation-related tumors or signaling pathway [11]. Lung cancer vasculature originates because of angiogenesis, vascular sheath growth, and endothelial progenitor cell growth [12]. Lung tissue hypoxia in the absence of vascularization results in tumor cells to produce lots of angiogenic factors inducing angiogenesis for tumor growth [13, 14]. Abnormality of Ang-2 expression has been reported to promote blood vessels and increase vascular permeability in ischemic and/or hypoxic environment [15, 16]. With the volume increasing, intratumoral hypoxia increased Ang-2 expression to promote angiogenesis for tumor metastasis [17, 18], and Ang-2 and Ang-2 mRNA in tissue or sera have been useful diagnostic biomarkers for lung cancer [19, 20]. However, the relationship between Ang-2 expression and proliferation, invasion, migration of cancer cells remains to be explored. Therefore, the objectives of this study were to investigate the alteration Ang-2 expression in lung tissues, and observe on effects of biological behaviors and epithelial-mesenchymal transition (EMT) abilities of lung cancer cells *in vitro* by silencing *Ang-2* with RNA interference technology.

## RESULTS

### Pulmonary Ang-2 expression and clinicopathological characteristics

**Figure 1:**
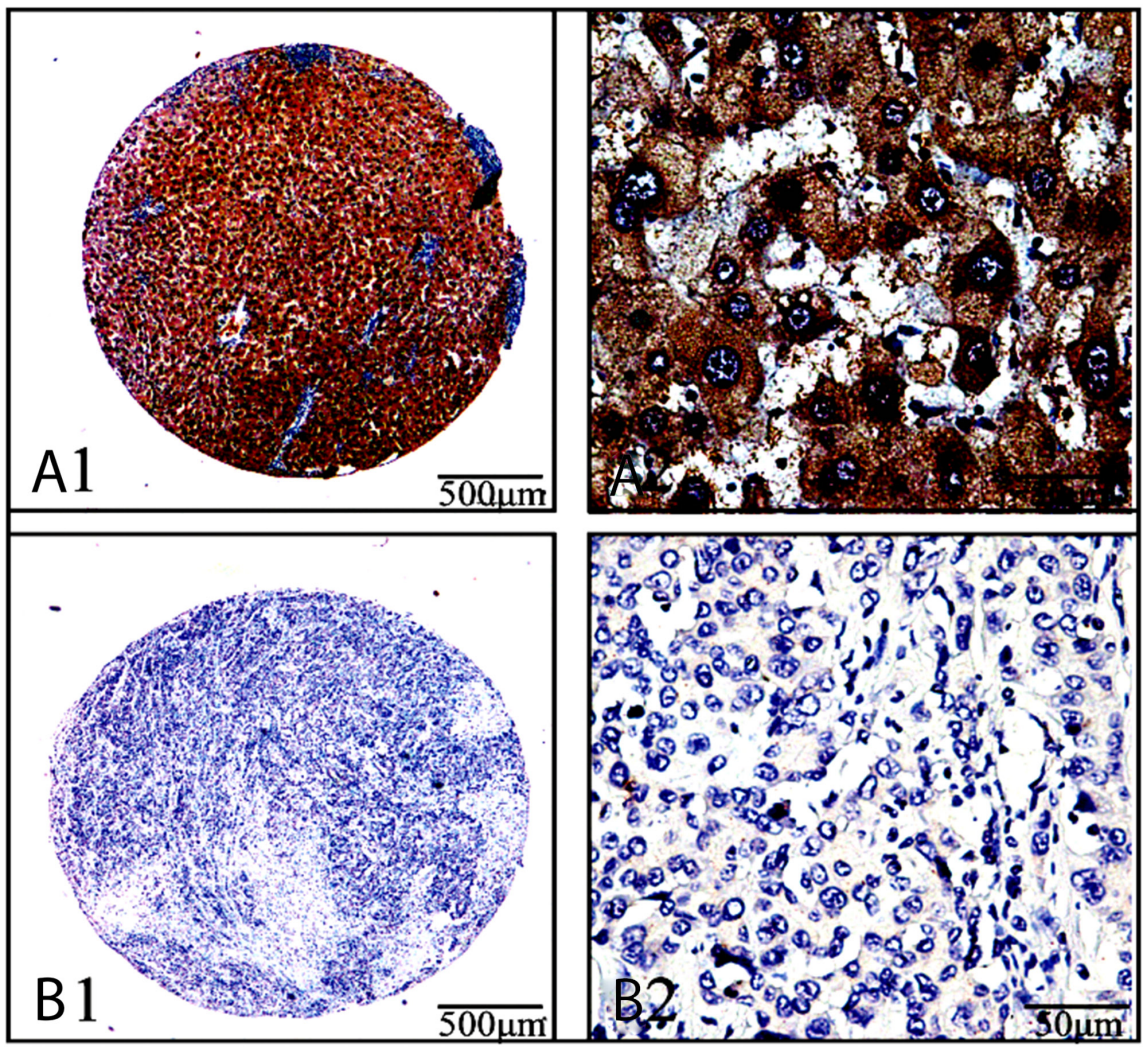
Ang-2 expression and cellular distribution by immunohistochemistryAng-2 expression in the cancerous-and their paracancerous-tissues of 122 patients with lung cancers were analyzed by immunohistochemistry with anti-human Ang-2 antibody (ab155106, Abcam, USA) **(A1 and A2)**, the stronger straining of Ang-2 expression in lung adenocarcinoma tissues, with deeper brown staining particles mainly localized in the cytoplasm and cell membranes; **(B1 and B2)**, the light straining of Ang-2 expression in their surrounding tissues; a1&b1, the original magnification × 40; a2&b2, the original magnification × 400.

Ang-2 expressions in 122 cases with lung cancers and their paracancerous tissues with the immunohistochemical analysis are shown in Figure 1. The Ang-2 expression with deeper brown staining particles in the cancerous tissues (Figure 1A1 and 1A2) were mainly localized in cytoplasm and cell membranes, and with lighter cytoplasm staining in their paracancerous tissues (Figure 1B1 and 1B2). Total incidence of Ang-2 expression in the cancerous group was up to 91.8 % (112 of 122) with significantly higher (χ^2^=103.753, *P*<0.001) than that in the surrounding group (27.9%, 34 of 122). The brown Ang-2 expressions were gradually increasing with clinical staging and showed very strong staining at advanced lung cancer. Correlation between Ang-2 levels and the clinicopathological parameters in cancerous tissues are shown in Table 1. Significant difference (χ^2^=57.254, *P*<0.001) of Ang-2 staining scores was found between cancerous tissues (71.3%, 87 of 122) with 2 ~ 3 scores and paracancerous tissues (23.0%, 28 of 122) with 0 ~ 1 scores. Clinicopathologic features of tumor derived Ang-2 expression was closely related to tumor diameter (χ^2^=7.883, *P*=0.005), degree of differentiation (χ^2^=4.554, *P*=0.033), TNM stage (χ^2^=5.039, *P*=0.025), and 5-year survival rate (χ^2^=11.220, *P*<0.001).

**Table 1:** Correlation of high Ang-2 expression with clinicopathologic characteristics of patients with lung cancer

Clinicopathologic characteristics	No. of patients (%)	Ang-2	%	χ^2^-value	*P*-value
		high (n)			
Lung cancer	122	87	71.3		
Gender				0.690	0.406
Male	59	40	67.8		
Female	63	47	74.6		
Age (years)				1.516	0.218
≤60	59	39	66.1		
>60	63	48	76.2		
Tumor diameter (cm)				7.883	**0.005**
≤2	31	16	51.6		
>2	91	71	77.0		
Differentiation				4.554	**0.033**
Well/Moderate	101	68	67.3		
Poor	21	19	90.5		
Lymph node metastasis				0.676	0.411
No	84	58	69.6		
Yes	38	29	76.3		
TNM staging				5.039	**0.025**
I-II	100	67	67.0		
III-IV	22	20	90.9		
Five-year survival				11.220	**<0.001**
Yes	42	22	52.38		
No	80	65	81.25		

**Ang-2**, angiopoietin-2; **TNM**, tumor node metastasis staging. Significant *P*-values (<0.05) are in bold.

### Univariate and multivariate analysis of Ang-2 expression

**Figure 2:**
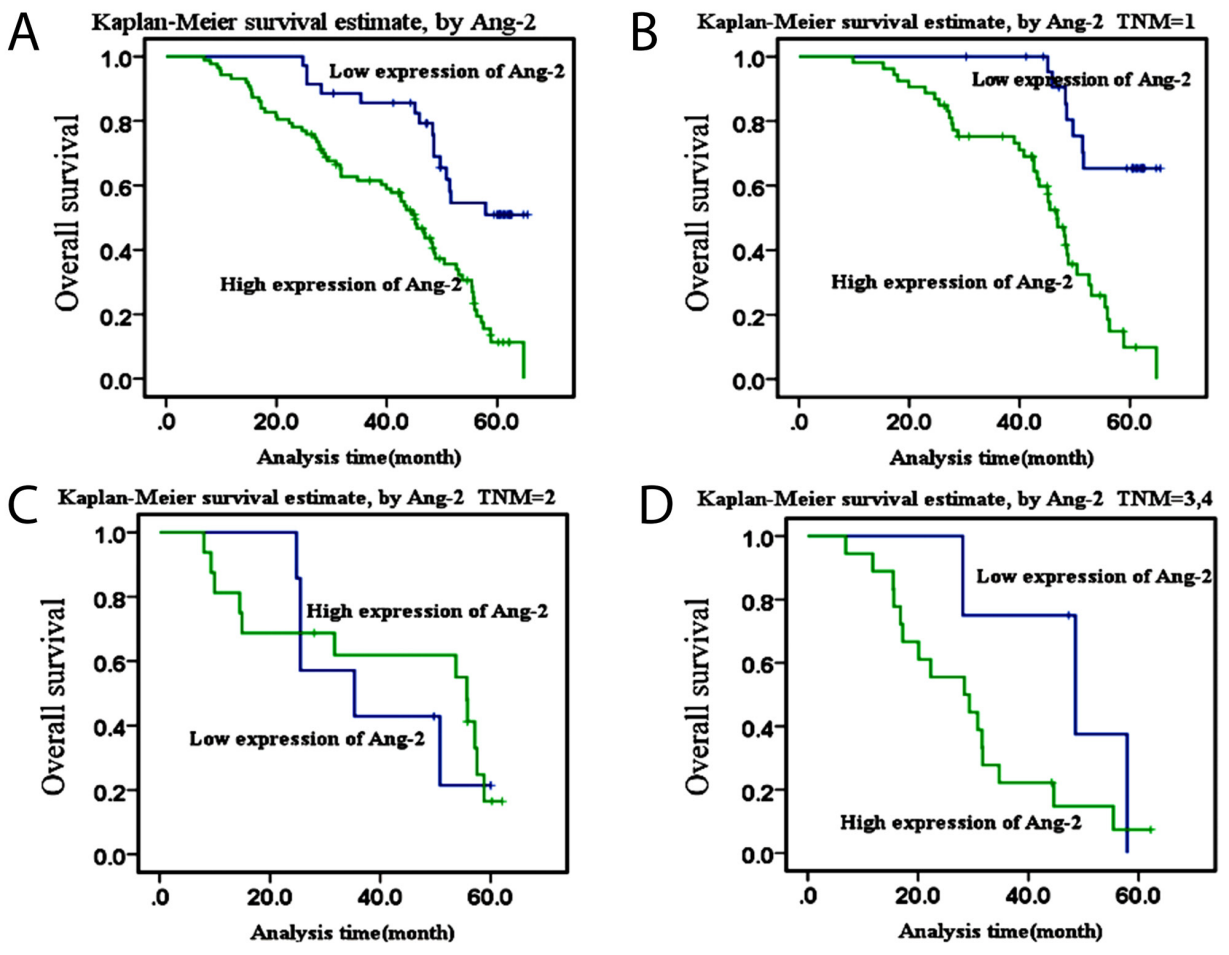
Kaplan-Meier survival curves of Ang-2 overexpressionThe survival curves of high Ang-2 expression in patients with lung cancer were made by the Kaplan-Meier method **(A)**, the overall survival curve of high Ang-2 expression in patients with lung cancer; **(B)**, the overall survival curve of high Ang-2 expression in patients with lung cancer at TNM stage I; **(C)**, the overall survival curve of high Ang-2 expression in patients with lung cancer at stage II; and **(D)**, the overall survival curve of high Ang-2 expression in patinets with lung cancer at stage III-IV, respectively.

Univariate and multivariate analysis of high Ang-2 expression in the tissues of lung cancer are shown in Table 2. Significant difference was found between high Ang-2 expression and 5-year survival rate of patients. The univariate analysis showed that Ang-2 expression (*P*<0.001), gender (*P*=0.042), differentiation degree (*P*<0.001), and TNM stage (*P*<0.001) were potential factors influencing survival of HCC patients. Further, the multivariate analysis showed that Ang-2 expression (*P*<0.001), differentiation degree (*P*=0.036), and TNM stage (*P*=0.030) were independent markers for HCC prognosis (Table 2). The Kaplan-Meier survival curves showed that the 5-year survival rate of high Ang-2 patients was significantly lower than that of cases with low or no expression (χ^2^=16.486, *P*<0.001; Figure 2A). However, significant difference was found only at TNM stage I (χ^2^=18.881, *P*<0.001; Figure 2B) but not at II (χ^2^ =0.81, *P*=0.776; Figure 2C) or III & IV (χ^2^=1.845, *P*=0.174; Figure 2D), indicated that the high Ang-2 level be an independent factor for poor outcome.

**Table 2:** Univariate and multivariate analysis of high Ang-2 expression in tissues of lung cancer

	Univariate			Multivariate		
	*P*	HR	95% CI	*P*	HR	95% CI
**Ang-2**						
High *vs* Low	**<0.001**	3.080	1.742-5.446	**<0.001**	3.144	1.738~5.689
**Gender**						
Male *vs* Female	**0.042**	1.578	1.017-2.450	0.082	1.515	0.948~2.421
**Age (years)**						
≤60 *vs* >60	0.628	1.115	0.717-1.733	0.746	0.927	0.585~1.448
**Diameter (cm)**						
≤2 *vs* >2	0.052	1.704	0.994-2.921	0.706	1.120	0.622~2.015
**Differentiation**						
Well/Moderate *vs* Poor	**<0.001**	0.347	0.203-1.593	**0.036**	0.490	0.252~0.944
**Lymph node metastasis**						
No *vs* Yes	0.081	1.497	0.952-2.355	0.497	0.806	0.432~1.503
**TNM staging**						
I/II *vs* III/IV	**<0.001**	1.690	1.283-2.228	**0.030**	1.531	1.041~2.252

**Ang-2**, angiopoietin-2; **CI**, confidence interval; **HR**, Haz ratio; **TNM**, tumor node metastasis staging; Significant *P*-values (<0.05) are in bold.

### Difference of lung cancer cell Ang-2 expression

**Figure 3:**
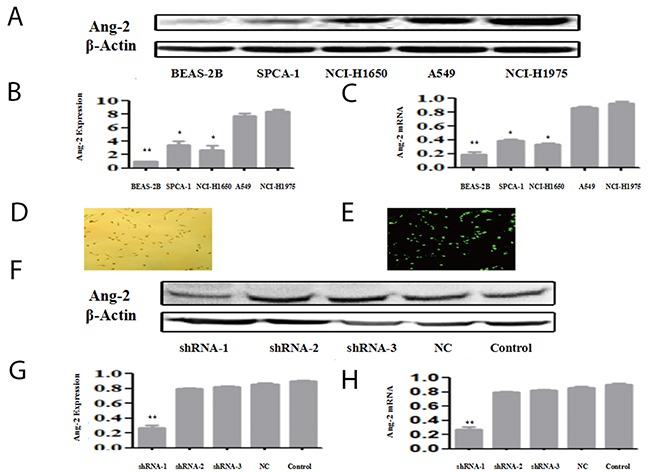
Ang-2 expression, gene transcription and shRNA suppression Ang-2 expression and gene transcription in lung Beas-2B cell, lung cancer (SPC-A-1, NCI-1650, A549, and NCI-1975) cell lines were analyzed at protein- by Western blotting or at mRNA-level by qRT-PCR **(A)**, the Ang-2 expressions in different cells at protein level with β-actin as control; **(B)**, the ratios from Ang-2 to β-actin in different cells at protein level; **(C)**, the ratios from Ang-2 to GAPDH expression in different cells at mRNA level; **(D)**, the phase-contrast image (X100 magnification) with the Ang-2-shRNA1 successfully transfected into A549 cells at 24 h; **(E)**, the fluorescence image (X100 magnification) with the Ang-2-shRNA1 successfully transfected into A549 cells; **(F)**, the different alterations of Ang-2 expression with β-actin as loading control at 48 h; **(G)**, the ratios from Ang-2 to β-actin protein in different cell lines; and **(H)**, the similar alterations of Ang-2 normalized to GAPDH in different cell lines at mRNA level. **Ang-2**, angiopoietin-2; **Ang-2 Exp.,** angiopoietin-2 protein expression; **shRNA**, the Ang-2-shRNA transfection group; **NC**, the negative control group; and **Control**, the blank control group. ^*^P<0.05. ^**^P<0.001.

The comparative analysis of Ang-2 expressing differences among lung cancer cell lines is shown in Figure 3. Ang-2 expression in lung cancer (SPC-A-1, NCI-1650, A549, and NCI-1975) cells at protein-(Figure 3A and 3B) or mRNA-(Figure 3C) level was significantly higher (1.5~4 times) than that in lung Beas-2B cells, especially in overexpression of A549 and NCI-H1975 cells. Furthermore, Ang-2-shRNAs and a negative-control shRNA (NC-shRNA) were successfully transfected into A549 cells, and fluorescence photomicrographs were observed at 24h (Figure 3D and 3E); Ang-2 at protein level at 48 h showed the different silencing efficiencies (Figure 3F) with high efficiency in shRNA-1, low efficiency in shRNA-2 and shRNA-3 (Figure 3G). Similar interference effects were observed at Ang-2 mRNA level (Figure 3H), showed that the most effective shRNA-1 could significantly downregulate *Ang-2* expression at mRNA or protein level.

### Ang-2 affected biological behaviors & EMT

**Figure 4:**
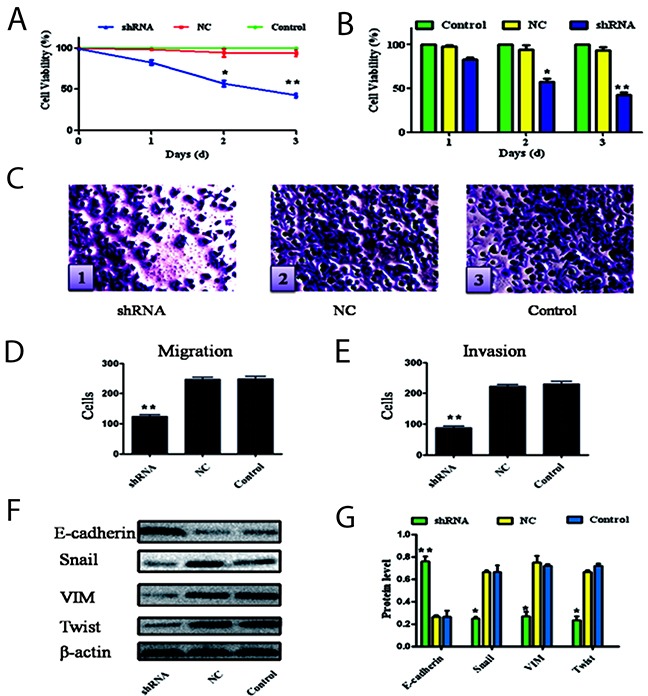
Silencing Ang-2 on effect of biological behaviors and EMT of cells The possibilities of cell proliferation, migration, invasion and EMT of the A549 cells were altered after the most effective Ang-2-shRNA-1 transfection. **(A & B)**, the cell proliferation ability was examined using CCK-8 at 450 nm. **(C)**, the cell patterns (×200 magnification) with the crystal violet staining solution: **c1**, the cells in the shRNA group; **c2**, the cells in the NC group, and **c3**, the cells in the control group; **(D)**, the cell migration abilities were presented as total number of cells that migrated to the bottom chamber without or with the transwell-precoated matrigel, as calculated at 6 random fields; **(E)**, the cellinvasion abilities were presented as total number of cells that migrated to the bottom chamber without or with the transwell-precoated matrigel, as calculated at 6 random fields; **(F)**, the EMT-related epithelial indicators and mesenchymal markers after silencing *Ang-2* transcritpion of A549 cells were analyzed by the Western blotting with β-actin as loading control; and **(G)**, the relative ratio from each protein to β-actin (n=3) compared with the control group. **Ang-2**, angiopoietin-2; **shRNA**, the Ang-2-shRNA transfection group; **NC**, the negative control group; and **Control**, the blank control group; ^*^P<0.05. ^**^P<0.001.

After the A549 cells with the most effective Ang-2-shRNA-1 transfection above, the abilities of cell proliferation, migration, invasion and EMT are shown in Figure 4. After transfected with shRNA-1 for 48 h, the cells showed lower proliferate abilities than those in the NC-or control-group at a time-dependent manner (Figure 4A and 4B). The abilities of the A549 cell migration and invasion migrated through the membrane in the migration chamber without (the shRNA group, shRNA, Figure 4C1) or with the transwell-precoated Matrigel lower than those in the negative control group (NC, Figure 4C2) or in the blank control group (control, Figure 4C3). The quantitative comparison of down-regulating Ang-2 on effect of invasion and migration of A549 cells are summarized in Table 3. Significant statistical difference (Figure 4D, migration: *t*=155.449, *P*<0.001; Figure 4E, invasiveness: *t*=142.933, *P*<0.001) was found between the shRNA-1- and the control-group, and silencing *Ang-2* transcription remarkably decreased the abilities of proliferation, migration and invasion of A549 cells.

**Table 3:** Silencing *Ang-2* transcritpion on effect of invasion and migration of lung cancer A549 cells

Group	Invasiveness			Migration		
	Mean ± SD	*t*-value	*p*-value	Mean ± SD	*t*-value	*p*-value
shRNA	96.39±18.831^*^	142.933	**<0.001**	126.67±18.279^*^	155.449	**<0.001**
NC	232.71±26.505^#^	1.630	0.103	249.22±19.187^#^	1.855	0.064
Control	234.01±25.120			250.40±23.203		

^*^P<0.001, the transfection group compared with the control group, according to cell count with crystal violet staining for invasion and migration analysis of A549 cells; Significant *P*-values (<0.001) are in bold.

**^#^*P*>0.05**, no significant difference was found bewteen the NC group and the control group; **shRNA**, the Ang-2-shRNA transfection group; **NC**, the negative control group; **Control**, the blank control group (n = 6); **SD**, standard deviation.

Above the basis of Ang-2 promoting lung cancer A549 cell migration and metastasis, the alterations of the EMT-related epithelial indicator (E-cadherin), mesenchymal marker (Vimentin, VIM), and transcriptional factors (Snail and Twist) expression that involved in lung cancer development were analyzed in Figure 4F and 4G. when lung cancer A549 cells with stable silencing *Ang-2* transcription in the shRNA group, the level of the epithelial E-cadherin expression was significantly (*P*<0.001, Figure 4F) increasing than that in the NC or control group; otherwise the levels of mesenchymal VIM, and transcriptional factor Snail and Twist expressions were significantly (*P*<0.05, Figure 4G) decreasing than those in the NC or control group.

## DISCUSSION

Lung cancer is the most common malignant tumors with and complex pathogenesis [2]. Its occurrence, development, and metastasis are associated with angiogenesis formation that make cancerous cells more invasive and affect chemotherapy and radiotherapy. Angiogenesis is a fundamental process involving a variety of pathological processes and sustains the progression of many neoplastic diseases [6]. Moreover, it may enhance cancer cell proliferation and anti-apoptosis, and facilitate metastasis [21–23]. Ang-2 upregulated in multiple inflammation-related tumors and associated closely with lung cancer progression. Although abnormal Ang-2 expression has been reported as useful biomarker for patients with lung cancer [24], however, the relationship between Ang-2 and tumor metastasis still needs to be identified. Therefore, the aims of this present study were to investigate the Ang-2 expression and its clinicopathological characteristics in tumor tissues, and silenced *Ang-2* transcription by specific shRNA to analyze the metastasis mechanism and biological behaviors of lung cancer.

The instability structure of tumor vascular, extreme hypoxia and drug delivery problems, these changes promote tumor progression, invasion of surrounding tissue, and distant metastasis [25]. Tumor tissue hypoxia causes cancer cells and macrophages to produce a large number of angiogenic factors that induce angiogenesis for the growth and development of lung cancer [26]. The expression of Ang-2 was increased in the peritumoral vascular remodeling area when capillary without formation, and might be involved in the initiation and regulation of lung cancer angiogenesis [27]. In this study, the level of Ang-2 expression in lung cancer tissues was significantly higher than that in their paracancerous tissue (Figure 1), and closely positive correlated with tumor size, TNM staging, poor differentiation, and 5-year survival rate (Figure 2), but there was no obvious relationship with patients’ age and lymph node metastasis (Tables 1&2), indicated that high Ang-2 expression could be an independent predictor related to distant metastasis for patients’ poor prognosis [28, 29].

When lung cancer cells proliferate, tumor volume and oxygen consumption increase significantly, the cancer cells become hypoxic and overexpress HIF-1α, which leads to the secretion of angiogenic factors (Ang-2, VEGF and so on) and induces angiogenesis [30, 31]. HIF-1α is suggested to be an important upstream molecule mediating VEGF expression and angiogenesis [16, 25]. However, it remains partially unclear that Ang-2 effects on the progression of lung cancer. In this study, the best inhibitory effect of shRNA sequence was screened through transfecting over-expression Ang-2 cell lines (Figure 3). Ang-2 expression in lung cancer (SPC-A-1, NCI-1650, A549, and NCI-1975) cells at protein or mRNA level was significantly higher than that in Beas-2B cells, especially in A549 cells. Among the shRNAs transfected into A549 cells, the effective shRNA-1 with significantly down-regulating *Ang-2* and inhibiting cell proliferation was chosen to analyze the biological behaviors of lung cancer cells.

Metastasis progress of lung cancer consists of distinct steps, including tumor angiogenesis, cell detachment, intravasation, micrometastasis formation, and growth [32–34]. After transfected with shRNA-1, the A549 cells showed lower proliferative abilities than those in the NC-or the control-group at a time-dependent manner (Figure 4). The abilities of the A549 cell migration and invasion in the shRNA group were lower than those in the NC-or control-group. Down-regulating Ang-2 affected the invasion and migration of A549 cells (Table 3), the data demonstrated that silencing *Ang-2* transcription could remarkably alter the biological behaviors with decreasing migration and invasion abilities of lung cancer cells.

EMT is a process that increases the metastasis and invasive potential of tumor cells, with losing epithelial cell characteristics and acquiring a mesenchymal pheno-type, including decreasing epithelial markers (cytokeratin or E-cadherin) and up-regulating mesenchymal indicators (VIM or N-cadherin) or transcription factors (Twist, Snail) [35–37]. Usually, inhibiting E-cadherin can induce invasiveness-related N-cadherin expression [38, 39]. VIM may enhance migration and invasiveness and related to reduce E-cadherin and upregulating N-cadherin, while increased VIM is correlated with poor prognosis [20, 40]. However, there have been no studies in which the expression of both EMT markers (Twist and VIM) was evaluated in lung cancer. In the current study, the alterations of the EMT-related E-cadherin, VIM, Snail, and Twist level were analyzed (Figure 4) and showed that the A549 cells with stable silencing *Ang-2* in the shRNA group, E-cadherin or signaling of VIM, Snail, and Twist were significantly increasing or decreasing than those in the NC or control group, indicated that abnormal Ang-2 expression promote lung cancer metastasis cascade through EMT formation.

**Figure 5:**
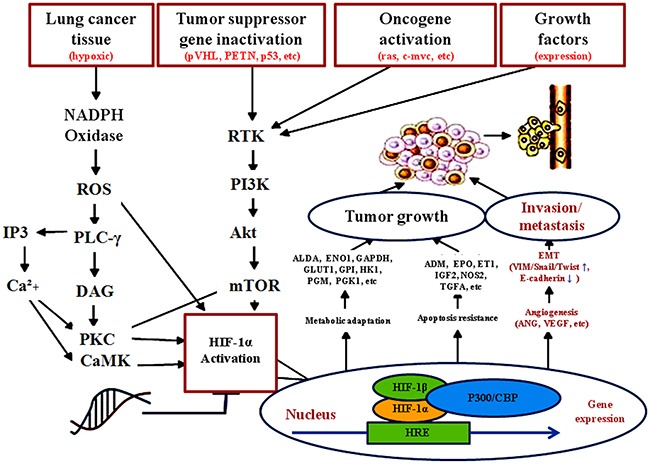
Possibility mechanism of tumor derived Ang-2 up-regulating EMT and facilitating lung cancer metastasis Ang-2 has been implicated in mediating inflammatory processes, and upregulated in multiple inflammation-related tumors or signaling pathway. With lung cancer volume increasing, intratumoral hypoxia in the absence of vascularization resulted in tumor cells to express Ang-2 inducing angiogenesis for tumor growth and increasing vascular permeability. Overexpression of Ang-2 facilitating proliferation, invasion, metastasis, and EMT of lung cancer cells that could be a new mechanism insight into tumor metastasis by inhibiting E-cadherin and upregulating VIM, Twist and Snail signaling. **Ang**, angiopoietin; **Ang-2**, angiopoietin-2; **CaMK,** calcium/calmodulin-dependent protein kinase; **EMT**, epithelial-mesenchymal transition; **ENO1**, enolase 1; **EPO**, erythropoietin; **ET1**, endothelin-1; **GAPDH**, glyceraldehyde-3-phosphate dehydrogenase; **Glut1**, glucose transporter 1; **HIF-1α**, hypoxia-inducible factor-1α; **HK1**, hexokinase 1; **HRE**, hypoxia-response element; **IGF2**, insulin-like growth factor 2; **IP3**, inositol 1,4,5-trisphate; **mTOR**, mechanistic target of rapamycin kinase; **NOS2**, nitric oxide synthase 2; **Oncogenes**, ras, c-myc etc; **PLC-γ**, phospholipase C gamma; **PI3K,** phosphatidylinositol 3-kinase; **PKC**, protein kinase C; **RTK,** receptor tyrosine kinase; **TGFA**, transforming growth factor alpha; **tumor suppressor genes**, pVHL, P_53_, PTEN, etc. **VEGF**, vascular endothelial growth factors; **VHL**, von Hippel-Lidau; **VIM**, Vimentin.

In conclusion, the abnormal Ang-2 expression promoted cell proliferation and affected on abilities of invasion, metastasis, and EMT of lung cancers that should provide a new mechanism insight into tumor metastasis by inhibiting E-cadherin and upregulating VIM, Twist and Snail signaling (Figure 5). Ang-2 was specifically overexpressed in tissues of lung cancers but not in benign lung diseases, and silencing *Ang-2* transcription by specific shRNA or anti-human Ang-2 antibodies could effectively inhibit tumor growth or metastasis with related EMT formation. However, there was a certain limitation that a relatively small sample of patients was involved in this study. Although the associations between Ang-2 and tumor metastasis were discovered, it was necessary to conduct more *in vitro* and *in vivo* assays to explore the exact function and mechanisms [41]. Further work should be explored combination of specific shRNA plus multi-targeting strategies for effective lung cancer therapy [42, 43].

## MATERIALS AND METHODS

### Lung tissues

For this study, fresh lung cancerous- and self-controlled paracancerous-tissues were obtained from 122 lung cancer patients who under-went surgery at the Affiliated Hospital of Nantong University, China, from Jul. 2009 to Dec. 2011. All cases of lung cancer were confirmed clinical and pathological evidence. All patients had not received radiotherapy or chemotherapy before operation. Lung specimen were collected from cases (59 men and 63 women) diagnosed with adenocarcinoma according to histological classification. Tumor node metastasis (TNM) staging of the patient cohort included 83 cases at stage-I, 17 with II, and 22 with III-IV. The tissue specimen were immediately frozen in liquid nitrogen and kept at −80°C until required. Each of the specimen was divided into two parts: one was used for immunohisto-chemical (IHC) staining and the other was fixed in formalin, embedded in paraffin, and then cut into 4-μm-thick section for pathological examination with hematoxylin & eosin (H&E) staining. This present study was approved by the Ethics Committee permission (TDFY2011008) of the Affiliated Hospital of Nantong University, and all patients signed informed consent. The related important clinical information of each patient was collected from their medical records.

### Cell lines and cell culture

Human lung epithelial Beas-2B cell line, non-small cell lung cancer (NSCLC) NCI-H1650, SPC-A-1, A549, and NCI-H1975 cell lines were obtained from the Institute of Biochemistry and Cell Biology, Chinese Academy of Science, Shanghai, China. All cell lines were cultured in RMPI-1640 medium (Corning, USA) containing 10 % fetal bovine serum (FBS, BI, Israel) and maintained in a 5% CO_2_ humidified atmosphere at 37°C. The expression and gene transcription of Ang-2 in human lung Beas-2B cells, lung cancer SPC-A-1, NCI-1650, A549, and NCI-1975 cell lines were analyzed at protein level by Western blotting and at mRNA level by quantitative real-time reverse transcription PCR (qRT-PCR).

### Western blotting

Phenylmethyl sulfonyl fluoride (PMSF), Radio Immunoprecipitation Assay (RIPA) lysis buffer, and Bicinchoninic Acid (BCA) Protein Assay kit were obtained from the Beyotime Institute of Biotechnology, Shanghai, China. Cells were harvested and washed with cold PBS twice, then lysed on ice in RIPA lysis buffer (1×PBS, 1% NP-40, 0.1% sodium dodecylsulfate (SDS), 5 mM EDTA, 0.5% sodium deoxycholate, and 1 mM sodium orthovanadate) that contained 100 μg/mL PMSF and protease inhibitors (KeyGen, Nanjing, China).

The total protein concentration of cells was determined by BCA method. Equivalent amounts (50 μg/per lane) from each sample was separated using a 10% SDS-polyacrylamide gels at 80 V for 40 min, then 120 V for 1h, and finally the gel was transferred to polyvinylidine difluoride (PVDF) membranes (Millipore Corporation, USA) at 300 mA for 120 min, blocked in 5% bovine serum albumin (BSA) in blocking buffer (Solarbio, China), and incubated with the primary antibodies rabbit anti-human Ang-2 (ab155106, diluted 1:1000; Abcam, UK), or anti-E-Cadherin (ab1416, diluted 1: 200; Abcam, UK), or anti-Vimentin (VIM, ab20346, diluted 1:2000; Abcam, UK), anti-Snail (ab167609, diluted 1:500; Abcam, UK), anti-Twist (ab50887, diluted 1:200; Abcam, UK), and β-actin (1:1000 dilution, CST, USA) overnight at 4°C, followed by incubation with secondary antibody horseradish peroxidase-conjugated (HRP)-conjugated goat anti-rabbit antibody (1:1000, Abbkine, China) at room temperature for 2h. Bands were visualized using an enhanced chemiluminescence system (ECL, Beyotime Institute of Biotechnology, China). The image was taken by the Quantity One software (Bio-Rad, Laboratories, Inc., USA).

### Total RNA extraction and qRT-PCR

Total RNAs from different cell lines were extracted using Trizol reagent (Invitrogen, USA). Reverse transcription to cDNA was carried out by using a Revert AidTM First Strand cDNA synthesis kit (MBI Fermentas, Vilnius, Lithuania USA) according to manufacturer’s instructions. In brief, in a 20 μL reaction mixture containing 2 μg RNA and oligo primers, at 42°C for 1 h, and synthesized cDNA was used for PCR by using SYBR®Premix Ex TaqTMII (TaKaRa, Dalian, China) with primers. The primers used for real-time RT-PCR purchased from Genechem Co., LUd (Nanjing, China) were as follows: Ang-2 forward, 5′-ACGGCTGTGATGATAGAAATAGG-3′ and reverse, 5′ GTAGTTGGATGATGTGCTTGTC-3′; glyceraldehyde-3-phosphate dehydrogenase (GAPDH): forward, 5′-CAAGGTCATCCATGACAACTTTG-3′; and reverse: 5′-GTC CACCACCCTGTTGCTGTAG-3′ (NM_017008). Cycling conditions were as follows: 2 min of preincubation at 95°C, 10s of denaturation at 95°C, 30s of annealing at 60°C, and 45s of extension at 72°C for 40 cycles with iCycler (Bio-Rad. USA). The fluorescent product was detected at the end of each cycle. Melting curves analysis was performed to determine PCR efficiency and purity of the amplified product after completed qRT-PCR, collecting fluorescence between 70°C and 95°C at 0.5°C increments. For data analysis, the raw threshold cycle (C_t_) value was normalized to the GAPDH for each sample to get ΔC_t_. The normalized ΔC_t_ was then calibrated to control cell samples to get ΔΔC_t_. The relative quantitative results of mRNA was calculated by the equation 2^−ΔΔCt^(n=3).

### Transfection of specific shRNA plasmids

Three different shRNA sequences of human Ang-2 and one negative control shRNA were designed and obtained from the Biomics BioUechnologies Co., LUd (Nantong, China). Their sequences were as follows: shRNA-1, 5′-TTACTCATTGTATGAACA T-3′; shRNA-2, 5′-CTAATTCTACAGAAGAGAT-3′, shRNA-3, 5′-CAC GGTGAATAATTCAGTT-3′, and a negative control shRNA (scramble), 5′-TTCTCCGAACGTG TCACGT-3′. Entranster™-D4000 (Engreen Biosystem Co, Ltd China) was used for transfection of cells according to manufacturer’s preoptimized instructions which may obtain maximal efficiency post-transfection.

In short, cells were seeded in 6-well plates at a density of 4 × 10^5^ cells/well, so that they could become about 60% confluence the next day. Transfection of shRNA plasmids according to manufacturer’s instructions, the one of the most effective silencing sequences were screened according to Ang-2 expression at protein level by Western blotting or at mRNA transcriptional level by qRT-PCR analysis.

### Cell proliferation assays

For analysis of cell proliferation, lung cancer A549 cells with Ang-2 overexpression divided into three groups: Ang-2-shRNA1 (shRNA1), negative-shRNA control (NC), and blank control group (Control) with total 5×10^3^ cells in 100μL of medium into 96-well plates (n=3/per group). Cell Counting Kit-8 (CCK-8, DOJINDO, Japan) was used to evaluate cell proliferation according to the manufacturer’s instructions. In brief, 10μL of CCK8 solution was added to the culture medium in each well and incubated for 2h. The cell absorbance at 450nm after 0h, 24h, 48h and 72h was determined, and the assays were repeated three times with triplicate samples.

### Transwell migration and invasion assays

For cell invasion assays, modified Boyden Chambers consisting of Transwell-precoated Matrigel membrane filter inserts with 8 μm pores were used in 24-well tissue culture plates (BD Biosciences, Bedford, MA). Cells (5 × 10^3^) from the shRNA, NC, and control groups (n=3/per group) were plated onto the top of the chamber in 100 μL of RMPI1640 medium without FBS and the bottom chamber was filled with RMPI1640 medium containing 10% FBS as a chemoattractant. After 24h of incubation in a 5% CO_2_ humidified chamber at 37°C, non-invading cells were removed by wiping the upper surface of the membrane with a cotton swab, and the filter membrane was fixed with 4% paraformaldehyde and stained with Crystal Violet Staining Solution. The degree of invasion was quantified by counting the cells that had migrated through the membrane in at least six random fields (total magnification, ×200) per filter. Experiments were repeated three times in triplicate. For analysis of cell migration, we use the modified Boyden Chambers without the Transwell-precoated Matrigel membrane filter with the method performed as above.

### Immunohistochemistry (IHC)

TMA slides used for IHC analysis were deparaffinized, and eroxidase was quenched with methanol and 3% H_2_O_2_ for 15 min. For antigen retrieval, the sections were boiled under pressure in citrate buffer (pH 6.0) for 3 min and then incubated for 120min with primary mouse anti-human Ang-2 (ab155106, Abcam, USA), washing with phosphate buffered saline (PBS), incubated with horse reddish peroxidase (HRP)-conjugated goat anti-mouse IgG (A21020; Abbkine, USA) for 15 min at 1:5000 dilution, and washed again with PBS. In finally, the slides were incubated with diaminobenzidine and counterstained with hematoxylin solution, dehydrated in ethanol, cleared in xylene, and cover-slipped. For the negative control reactions, the primary antibody was instead with PBS.

### Evaluation of IHC findings

The results of IHC staining were assessed by two independent pathologists without knowledge of the clinicopathologic features. The percentages for Ang-2 positive cells were scored as follows: 0 (0%), 1 (1% ~ 33%), 2 (34% ~ 66%), and 3 (67% ~ 100%) according to the previous reported method [44]. Staining intensity was stratified into four categories: 0 (negative), 1 (weakly), 2 (moderate), and 3 (strongly). The sum of the percentage and intensity score was defined as the IHC staining score. According to above criterion, the lung tissues with Ang-2 expression were divided into two groups: low with 0 ~ 1 scores and high 2 ~ 3 scores. The higher score for positive percentages and staining intensity was taken as the final score when there was a difference between duplicate tissue scores.

### Statistical analysis

Statistical analysis was carried out by using SPSS software (version 20.0). Results of cell proliferation, transwell migration and invasion were expressed as the mean ± standard deviation (SD). Difference between groups or clinicopathological factors was evaluated by the χ^2^ or *t* test. Survival curves were made using the Kaplan-Meier method and analyzed by the log-rank test or the Cox regression. Significance level was set at *P* value < 0.05 for all tests.

## Abbreviations

Ang-2: Angiopoietin-2
Ang-2-shRNA: Ang-2-short hairpin RNA
VEGF: vascular endothelial growth factor
TNM: tumor node metastasis staging
EMT: epithelial-mesenchymal transition
RNAi: RNA interference
IHC: immunohistochemistry

## References

[1] Chen W, Zheng R, Zhang S, Zeng H, Zuo T, Xia C, Yang Z, He J (2017). Cancer incidence and mortality in China in 2013: an analysis based on urbanization level. Chin J Cancer Res.

[2] Malvezzi M, Carioli G, Bertuccio P, Boffetta P, Levi F, La Vecchia C, Negri E (2017). European cancer mortality predictions for the year 2017, with focus on lung cancer. Ann Oncol.

[3] Cassidy RJ, Zhang X, Patel PR, Shelton JW, Escott CE, Sica GL, Rossi MR, Hill CE, Steuer CE, Pillai RN, Ramalingam SS, Owonikoko TK, Behera M (2017). Next-generation sequencing and clinical outcomes of patients with lung adenocarcinoma treated with stereotactic body radiotherapy. Cancer.

[4] Brock M, Mei Y (2017). Protein functional effector sncRNAs (pfeRNAs) in lung cancer. Cancer Lett.

[5] Lu H, Jiang Z (2017). Advances in antiangiogenic treatment of small-cell lung cancer. Onco Targets Ther.

[6] Zhou L, Lv T, Zhang Q, Zhu Q, Zhan P, Zhu S, Zhang J, Song Y (2017). The biology, function and clinical implications of exosomes in lung cancer. Cancer Lett.

[7] Sanmartín E, Sirera R, Usó M, Blasco A, Gallach S, Figueroa S, Martínez N, Hernando C, Honguero A, Martorell M, Guijarro R, Rosell R, Jantus-Lewintre E (2014). A gene signature combining the tissue expression of three angiogenic factors is a prognostic marker in early-stage non-small cell lung cancer. Ann Surg Oncol.

[8] Honguero Martínez AF, Arnau Obrer A, Figueroa Almánzar S, León Atance P, Guijarro Jorge R (2014). Analysis of expression of vascular endothelial growth factor A and hypoxia inducible factor-1alpha in patients operated on stage I non-small-cell lung cancer. Lung Cancer Int.

[9] Gerald D, Chintharlapalli S, Augustin HG, Benjamin LE (2013). Angiopoietin-2: an attractive target for improved antiangiogenic tumor therapy. Cancer Res.

[10] Yoh K, Goto Y, Naito Y, Kishi K, Mori K, Hotta K, Hosomi Y, Yamada K, Tanai C, Tomizawa Y, Inoue A, Hasegawa Y, Nishio M (2017). Impact of maintenance therapy for patients with non-small cell lung cancer in a real-world setting. Anticancer Res.

[11] Scholz A, Plate KH, Reiss Y (2015). Angiopoietin-2: a multifaceted cytokine that functions in both angiogenesis and inflammation. Ann N Y Acad Sci.

[12] Andersen S, Donnem T, Al-Shibli K, Al-Saad S, Stenvold H, Busund LT, Bremnes RM (2011). Prognostic impacts of angiopoietins in NSCLC tumor cells and stroma: VEGF-A impact is strongly associated with Ang-2. PLoS One.

[13] Coffelt SB, Tal AO, Scholz A, De Palma M, Patel S, Urbich C, Biswas SK, Murdoch C, Plate KH, Reiss Y, Lewis CE (2010). Angiopoietin-2 regulates gene expression in TIE2-expressing monocytes and augments their inherent proangiogenic functions. Cancer Res.

[14] Benest AV, Kruse K, Savant S, Thomas M, Laib AM, Loos EK, Fiedler U, Augustin HG (2013). Angiopoietin-2 is critical for cytokine-induced vascular leakage. PLoS One.

[15] Semenza GL (2013). Cancer-stromal cell interactions mediated by hypoxia-inducible factors promote angiogenesis, lymphangiogenesis, and metastasis. Oncogene.

[16] Yang XL, Wang L, Sai WL, Cai Y, Gu JJ, Chen X, Yao DF (2016). Clinicopathological features of hypoxia-inducible factor-1α expression in patients with non-small cell lung cancer. Oncol Transl Med.

[17] Albini A, Noonan DM (2012). Angiopoietin2 and tie2: tied to lymphangiogenesis and lung metastasis. New perspectives in antimetastatic antiangiogenic therapy. J Natl Cancer Inst.

[18] Xuan ZX, Zhang S, Yuan SJ, Wang W, Yu J (2016). Prognostic value of angio-poietin-2 in non-small cell lung cancer patients: a meta-analysis. World J Surg Oncol.

[19] Xu C, Wang W, Wang Y, Zhang X, Yan J, Yu L (2016). Serum angiopoietin-2 as a clinical marker for lung cancer in patients with solitary pulmonary nodules. Ann Clin Lab Sci.

[20] Coelho AL, Araújo A, Gomes M, Catarino R, Marques A, Medeiros R (2014). Circulating Ang-2 mRNA expression levels: looking ahead to a new prognostic factor for NSCLC. PLoS One.

[21] Davies J, Patel M, Gridelli C, de Marinis F, Waterkamp D, McCusker ME (2017). Real-world treatment patterns for patients receiving second-line and third-line treatment for advanced non-small cell lung cancer: a systematic review of recently published studies. PLoS One.

[22] Göke A, Göke R, Ofner A, Herbst A, Lankat-Buttgereit B (2015). The FGFR inhibitor NVP-BGJ398 induces NSCLC cell death by activating caspase-dependent pathways as well as caspase-independent apoptosis. Anticancer Res.

[23] Huang Y, Song N, Ding Y, Yuan S, Li X, Cai H, Shi H, Luo Y (2009). Pulmonary vascular destabilization in the premetastatic phase facilitates lung metastasis. Cancer Res.

[24] Coelho AL, Araújo AM, Gomes MP, Catarino RJ, Andrade EB, Lopes AM, Medeiros RM (2015). Combined Ang-2 and VEGF serum levels: holding hands as a new integral biomarker in non-small-cell lung cancers. Future Oncol.

[25] Deng PB, Hu CP, Xiong Z, Yang HP, Li YY (2013). Treatment with EGCG in NSCLC leads to decreasing interstitial fluid pressure and hypoxia to improve chemo-therapy efficacy through rebalance of Ang-1 and Ang-2. Chin J Nat Med.

[26] Coffelt SB, Chen YY, Muthana M, Welford AF, Tal AO, Scholz A, Plate KH, Reiss Y, Murdoch C, De Palma M, Lewis CE (2011). Angiopoietin 2 stimulates TIE2-expressing monocytes to suppress T cell activation and to promote regulatory T cell expansion. J Immunol.

[27] Keskin D, Kim J, Cooke VG, Wu CC, Sugimoto H, Gu C, De Palma M, Kalluri R, LeBleu VS (2015). Targeting vascular pericytes in hypoxic tumors increases lung metastasis via angiopoietin-2. Cell Rep.

[28] Nasarre P, Thomas M, Kruse K, Helfrich I, Wolter V, Deppermann C, Schadendorf D, Thurston G, Fiedler U, Augustin HG (2009). Host-derived angiopoietin-2 affects early stages of tumor development and vessel maturation but is dispensable for later stages of tumor growth. Cancer Res.

[29] Oztutgan T, Demirer E, Tas D, Uysal A, Caliskan T, Kucukodaci Z, Ayten O, Okutan O, Kartaloglu Z (2016). A comparative analysis of angiopoietin 2 immunohisto-chemical staining in various stages of lung cancer. Niger J Clin Pract.

[30] Frezzetti D, Gallo M, Roma C, D’Alessio A, Maiello MR, Bevilacqua S, Normanno N, De Luca A (2016). Vascular endothelial growth factor a regulates the secretion of different angiogenic factors in lung cancer cells. J Cell Physiol.

[31] Secker GA, Harvey NL (2015). VEGFR signaling during lymphatic vascular development: from progenitor cells to functional vessels. Dev Dyn.

[32] Park JS, Kim IK, Han S, Park I, Kim C, Bae J, Oh SJ, Lee S, Kim JH, Woo DC, He Y, Augustin HG, Kim I (2016). Normalization of tumor vessels by Tie2 activation and Ang2 inhibition enhances drug delivery and produces a favorable tumor microenvironment. Cancer Cell.

[33] Rigamonti N, De Palma M (2013). A role for angiopoietin-2 in organ-specific metastasis. Cell Rep.

[34] Minami T, Jiang S, Schadler K, Suehiro J, Osawa T, Oike Y, Miura M, Naito M, Kodama T, Ryeom S (2013). The calcineurin-NFAT-angiopoietin-2 signaling axis in lung endothelium is critical for the establishment of lung metastases. Cell Rep.

[35] Nagaraja SS, Krishnamoorthy V, Raviraj R, Paramasivam A, Nagarajan D (2017). Effect of Trichostatin A on radiation induced epithelial-mesenchymal transition in A549 cells. Biochem Biophys Res Commun.

[36] Xu Y, Lou Z, Lee SH (2017). Arctigenin represses TGF-β-induced epithelial mesen-chymal transition in human lung cancer cells. Biochem Biophys Res Commun.

[37] Zhang Z, Yang Y, Zhang X (2017). MiR-770 inhibits tumorigenesis and EMT by targeting JMJD6 and regulating WNT/β-catenin pathway in non-small cell lung cancer. Life Sci.

[38] Bian T, Jiang D, Liu J, Yuan X, Feng J, Li Q, Zhang Q, Li X, Liu Y, Zhang J (2017). miR-1236-3p suppresses the migration and invasion by targeting KLF8 in lung adenocarcinoma A549 cells. Biochem Biophys Res Commun.

[39] Yun Y, Gao R, Yue H, Guo L, Li G, Sang N (2017). Sulfate aerosols promote lung cancer metastasis by epigenetically regulating the epithelial-to-mesenchymal transition (EMT). Environ Sci Technol.

[40] Huang LX, Hu CY, Jing L, Wang MC, Xu M, Wang J, Wang Y, Nan KJ, Wang SH (2017). microRNA-219-5p inhibits epithelial-mesenchymal transition and metastasis of colorectal cancer by targeting lymphoid enhancer-binding factor 1. Cancer Sci.

[41] Holopainen T, Saharinen P, D’Amico G, Lampinen A, Eklund L, Sormunen R, Anisimov A, Zarkada G, Lohela M, Heloterä H, Tammela T, Benjamin LE, Ylä-Herttuala S (2012). Effects of angiopoietin-2-blocking antibody on endothelial cell-cell junctions and lung metastasis. J Natl Cancer Inst.

[42] Brustugun OT, Sprauten M, Helland A (2017). Real-world data on nivolumab treatment of non-small cell lung cancer. Acta Oncol.

[43] Thomas M, Kienast Y, Scheuer W, Bähner M, Kaluza K, Gassner C, Herting F, Brinkmann U, Seeber S, Kavlie A, Welschof M, Ries S, Weidner KM (2013). A novel angiopoietin-2 selective fully human antibody with potent anti-tumoral and anti-angiogenic efficacy and superior side effect profile compared to Pan-Angiopoietin-1/-2 inhibitors. PLoS One.

[44] Pan LH, Yao M, Cai Y, Gu JJ, Yang KL, Wang L, Yao DF (2016). Oncogenic Wnt3a expression as an estimable prognostic marker for hepatocellular carcinoma. World J Gastroenterol.

